# Drug-Target Interaction Prediction Based on Multisource Information Weighted Fusion

**DOI:** 10.1155/2021/6044256

**Published:** 2021-11-24

**Authors:** Shuaiqi Liu, Jingjie An, Jie Zhao, Shuhuan Zhao, Hui Lv, ShuiHua Wang

**Affiliations:** ^1^College of Electronic and Information Engineering, Hebei University, Baoding 071000, China; ^2^Machine Vision Technology Creation Center of Hebei Province, Baoding 071000, China; ^3^Beagledata Technology (Beijing) Co. Ltd., Beijing 100089, China; ^4^School of Architecture Building and Civil Engineering, Loughborough University, Loughborough LE11 3TU, UK

## Abstract

Recently, in most existing studies, it is assumed that there are no interaction relationships between drugs and targets with unknown interactions. However, unknown interactions mean the relationships between drugs and targets have just not been confirmed. In this paper, samples for which the relationship between drugs and targets has not been determined are considered unlabeled. A weighted fusion method of multisource information is proposed to screen drug-target interactions. Firstly, some drug-target pairs which may have interactions are selected. Secondly, the selected drug-target pairs are added to the positive samples, which are regarded as known to have interaction relationships, and the original interaction relationship matrix is revised. Finally, the revised datasets are used to predict the interaction derived from the bipartite local model with neighbor-based interaction profile inferring (BLM-NII). Experiments demonstrate that the proposed method has greatly improved specificity, sensitivity, precision, and accuracy compared with the BLM-NII method. In addition, compared with several state-of-the-art methods, the area under the receiver operating characteristic curve (AUC) and the area under the precision-recall curve (AUPR) of the proposed method are excellent.

## 1. Introduction

Targets refer to biological macromolecules which can specifically bind to small molecule compounds in the organism and produce specific physiological or pharmacological effects. They have the function of organism physiological accommodation or disease prophylaxis and treatment. The most common targets are ion channels, enzymes, receptors, and other molecules. Drug-target interaction prediction is widely used nowadays. Furthermore, it has important implications for elucidating the mechanism of drug molecules, which can be used for the manufacture of new drugs [1]. The essential step to new drug development is the discovery and repositioning of targets [2]. The procedure of searching new candidate drugs for known targets is called drug discovery, and the procedure of searching new targets for known drugs is called drug repositioning [3]. Only a small part of the interaction relationships in the dataset has been verified until now. Traditional biological testing requires a lot of investment, which greatly restricts the development of new drugs. Therefore, research related to drug-target interactions has turned into a hotspot in pharmaceutical sciences [4]. Due to advances in technology, the understanding of substances such as genes, proteins, cells, and so on has been deepened. With the assistance of computer technology, drug discovery has been greatly sped up. Moreover, it is helpful to shorten the development cycle of new drugs and reduce the cost of research and development [5].

Traditional drug-target interaction prediction methods are roughly split into docking simulation methods [6, 7] and ligand-based methods [8]. The former is based on the known three-dimensional structure of targets, which can predict the biological activity of candidate compounds by calculating the binding capacity of small molecules to targets in compound datasets [9]. However, when lacking the three-dimensional structure of targets, docking simulation methods do not work. At present, there are still numerous three-dimensional structures not being resolved, which makes docking simulation methods greatly limited [10]. The ligand-based methods mainly include chemical similarity search and reverse pharmacophore search. The former approach is based on the fact that drugs with approximate structures can interact with targets that have identical or approximate characters [11]. The latter method constructs a pharmacophore database containing multiple pharmacophore models in advance. Then, a single query molecule is used to reverse match. Finally, targets matching the query structure better will be found. The method based on the ligand structure will fail when a few ligands are learned. Therefore, the traditional calculation method depends heavily on the chemical structure of the drug, which has great limitations [12].

In recent years, scholars from all over the world have proposed some methods from all aspects for the study of drug-target interactions (DTIs), which greatly improved the prediction efficiency and accuracy. Compared with traditional methods, these methods make full use of computer technology to assist research, which is helpful for shortening the development cycle of new drugs and reducing research costs. The commonly used methods are mainly divided into four kinds, including prediction methods based on matrix decomposition, prediction methods based on network inference, prediction methods based on drug and target characteristics, and prediction methods based on the bipartite graph model. The mentioned method will be introduced in the following.

The method based on matrix decomposition predicted the relationships between drugs and targets by matrix decomposition. Gonen et al. [13] proposed a DTI prediction method based on kernelized Bayesian matrix factorization (KBMF), which combines kernel-based dimensionality reduction, matrix factorization, and binary classification to predict interactions. This method only needs to know the chemical similarity of drugs and the spatial similarity of targets and then uses variational inference to update the parameters. To promote the model effect, Liu et al. [14] proposed a DTI prediction method based on neighborhood regularized logistic matrix factorization (NRLMF). The difference between NRLMF and KBMF is that NRLMF assigns higher weights to the pairs that have known interactions. The Bayesian algorithm has many applications in the prediction of DTIs. Peska et al. [15] proposed a DTI prediction method based on the Bayesian ranking method. This algorithm utilized target deviation and structure similarity of drugs and targets to predict DTIs by combining Bayesian personalized ranking [16]. The Bayesian algorithm can also be used for the hyperparameter optimization of the matrix factorization method. Ban et al. [17] proposed an interaction prediction method derived from Bayesian optimization, which greatly reduces the calculation time of hyperparameter optimization.

Predicting the relationship derived from the network inference method mainly refers to constructing a heterogeneous network by using the similarity of drug to drug and similarity of target to target. Then, DTIs can be predicted based on the network. These kinds of methods can be split into three types: supervised, semisupervised, and unsupervised. Cheng et al. [18] proposed a supervised inference DTI prediction method. This algorithm only uses the topological similarity of the bipartite network, which is constructed by the relation of the drug and target, to infer the new targets of known drugs. The results proved that the capability of this method surpasses that of the DTI inference algorithms based on drug or target similarity. Pliakos et al. [19] combined supervised learning with multioutput tasks and regarded the prediction as a multioutput task by learning to reconstruct the biclustering tree on the network. Yan et al. [20] proposed a semisupervised DTI inference method to infer the label of drug nodes by using label propagation. Compared to the traditional supervised and semisupervised DTI inference methods, the network inference method based on the random walk framework proposed by Seal et al. [21] can update the labels in the heterogeneous network through the labels in the homogeneous network. It can utilize network data integration tools to predict the relationship. Therefore, network inference methods based on the random walk framework are often used for prediction. Thafar et al. [22] proposed a method for predicting the relationship by utilizing graph embedding, graph mining, and DTI prediction methods based on the similarity of drugsto targets. Zeng et al. [23] implemented arbitrary-order proximity network embedding on a heterogeneous network and used deep learning algorithms to predict DTIs. Samizadeh et al. [24] used a new method derived from node embedding and achieved the classification result with a binary classifier.

Most DTI prediction methods based on machine learning demand features of drugs and targets to predict the interaction [25, 26]. Among them, each drug-to-target pair is expressed by a feature vector with a certain length. The feature vector of the drug-to-target pair is divided into two types: interaction and noninteraction. To reduce the computational complexity, Van et al. [27] proposed a weighted nearest neighbor (WNN) method, which improved the Gaussian interaction profile (GIP) [28] method and used the nearest neighbor information to predict new drugs. Some scholars extract reliable negative samples from drug-to-target datasets and then combine them with positive samples on the original data to construct a classifier. For instance, Lan et al. [29] proposed a DTI prediction method by using positive samples and unlabeled samples. In this method, unknown interactions between drug-to-target pairs are regarded as unlabeled samples, and the weighted support vector machine (SVM) is used for DTI predicting. Peng et al. [30] proposed a negative sample extraction method to reduce the false-positive error caused by randomly selecting negative samples. In DTI prediction, most methods face two problems: class imbalance and high-dimensional data. Redkar et al. [31] solved the problem of high-dimensional data by efficiently and orderly encoding the target protein. The problem of class imbalance is solved by using synthetic minority oversampling.

Bleakley et al. [32] proposed bipartite local models (BLMs), which used known drugs and targets to train local models. Then, the final result was calculated by combining the prediction results of the two local models. The limitation of the BLM is that it is difficult to predict the interaction between drugs and targets when the interaction relationship is not known. For this reason, Mei et al. [33] proposed the BLM-NII method based on neighbor-based interaction profile inferring (NII). To further promote the prediction effect, Buza et al. [34] introduced a regression technique as a local model to predict the interaction and enhanced the representation of drugs and targets in the multimodal similarity space. Subsequently, Buza et al. [35] proposed a DTI prediction algorithm by using an asymmetric loss model based on BLM. The modified linear regression model (MOLIERE) improved the prediction capability of the BLM model. The abovementioned DTI prediction algorithms are based on the classifying method, which is all that is needed to perform feature extraction. When using a classifier, positive samples and negative samples are needed. However, in DTI prediction problems, samples with unknown labels are often regarded as negative samples, which will impact the results and have certain limitations.

In the existing research, most researchers do not know which interaction pairs are negative samples. However, there may be some drug-to-target pairs in unlabeled samples which have interactions but have not been verified by experiments. In this paper, these unknown interaction pairs are regarded as unlabeled samples. The unlabeled samples are screened by three methods: the drug similarity method, the random walk with restart method, and the WNN-GIP method. Then, the weighted fusion method of multisource information is used to fuse the screened results obtained by the three methods. Finally, the interaction matrix in the training set is revised according to the fusion results, and then, we utilize the BLM-NII model to predict interactions. Experiments show that the proposed method can obtain a superior prediction effect.

## 2. Related Algorithms

### 2.1. Random Walk

Graph is a kind of data structure which can be used to express the complex interactive relationship in the real world. Each graph has two basic components, namely, nodes and edges. Nodes are connected by edges. In terms of drug-to-target interaction prediction, drugs and targets are expressed by nodes and the relationship is expressed by edges. For the graph composed of drugs and targets, the random walk can be made on the graph so as to predict the interactions.

Random walk is a common method of information dissemination. The fundamental principle of the random walk is to walk from one vertex by traversing a graph. At each vertex, a random walker has two choices: one of the choices is to walk to the neighbor of this vertex with probability 1 − *a*, and the other is to skip to any vertexes randomly with probability *a*. This probability represents the possibility of a skip. After the walk, a probability distribution is obtained, from which we can get the probability of each node being visited. Then, we use this distribution as a starting probability and iterate this process. The distribution will stabilize when preconditions are reached. The random walk with a restart [36] is a kind of variant of the random walk. It starts from a certain node and faces two choices during each step of the walk, randomly selecting neighboring nodes or returning to the seed node with a certain probability. Compared to the traditional random walk, the random walk with restart can more fully explore the direct or indirect relationship between nodes.

**Figure 1:**
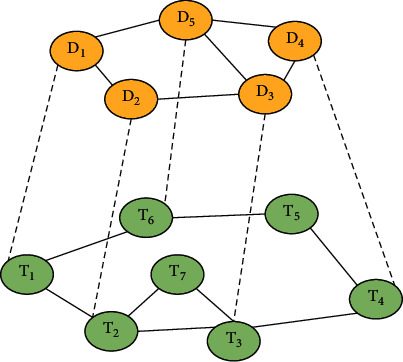
Drug-target interaction heterogeneous network.

For drug-target relationship prediction, the heterogeneous network is shown in Figure 1. The yellow part represents the drug similarity network, the green part represents the target similarity network, and the dotted line between the two networks represents the interaction relationship. Nodes receive the information of another homogeneous network through the heterogeneous network in the process of traveling, thus improving the initial label setting of drug and target homogeneous networks.

The random walk with a restart can effectively integrate the abovementioned networks into a framework. The constructed heterogeneous network does not depend on the three-dimensional structure information of the drug and target. However, it is known that the drug-to-target interactions only account for a small part, which leads to sparse interactions in heterogeneous networks. For sparse networks, new drugs or new targets are often isolated. It is difficult for us to predict the interaction, which also limits the improvement of random walk capacity. To promote the predictive power of the random walk, the multisource information fusion method can be used to select drug-to-target pairs with high interaction probability. Then, we add the selected drug-to-target pairs to the positive samples. Thus, more reliable drug-target interaction relations can be obtained, the sparsity of the network can be reduced, and isolated subnetworks can also be reduced.

### 2.2. WNN-GIP

In this paper, we assume that *K*_GIP,*d*_ represents the similarity matrix between drug and drug, *K*_GIP,*t*_ represents the similarity matrix between target and target, and *K*_chemical,*d*_ denotes the similarity of drug chemical structure. GIP [28] used Gaussian kernel function to express *K*_GIP,*d*_ and *K*_GIP,*t*_. The kernel function *K*_*d*_ can be obtained by combining *K*_GIP,*d*_ and *K*_chemical,*d*_ as

(1) $$\begin{array}{c} K_{d}=\alpha_{d}K_{\text{chemical,}d}+\left(1-\alpha_{d}\right)K_{\text{GIP},d}, \end{array}$$

where *K*_*d*_ represents the features of the drug.

Similarly, we can combine *K*_GIP,*t*_ with sequence similarity of protein gene *K*_genomic,t _ according to a certain weight *α*_*t*_ to obtain the kernel function *K*_*t*_, such as

(2) $$\begin{array}{c} K_{t}=\alpha_{t}K_{\text{genomic,t }}+\left(1-\alpha_{t}\right)K_{\text{GIP},t}, \end{array}$$

where *K*_*t*_ represents the features of the target.

Combining *K*_*d*_ and *K*_*t*_ by the Kronecker product, we can obtain a kernel matrix *K* about drug-target pairs. According to the combined kernel matrix *K* and the interaction profile *y*, the regularized least-squares classifier can be used to obtain the prediction value $\hat{y}$, and its calculation formula is shown in the following formula:

(3) $$\begin{array}{c} \hat{y}=K{\left(K+\sigma I\right)}^{-1}y, \end{array}$$

where *σ* represents a regularization parameter and *I* is the identity matrix.

GIP can only deal with drugs that have at least one known interaction. For new drugs, weighted nearest neighbor (WNN) information is used to predict drug interaction relationships, which is shown in the following formula:

(4) $$\begin{array}{c} y_{\text{WNN}}^{d}=\sum_{i=1}^{n_{d}}w_{i}y_{i}, \end{array}$$

where *n*_*d*_ means the number of drugs in the dataset, *w*_*i*_ means the weight, *y*_*i*_ denotes the row *i* of adjacency matrix *Y*, which represents the relationship between the drug *d*_*i*_ and all targets, and *y*_WNN_^*d*^ represents the predicted score of new drug *d*.

WNN infers the interaction of new drugs according to the interaction relationship in the dataset, and the prediction score is the weighted sum of all drug interactions. Among them, the weight is determined by how similar the current drug is to the new drug. The drug with high similarity to the new drug has high weight, while the drug with low similarity has low weight and makes little contribution to the final prediction results.

GIP is used to predict drugs with at least one known interaction, and WNN is used to predict new drugs. Combining the advantages of the mentioned two algorithms, WNN-GIP can be obtained to predict drug-target interactions. However, WNN-GIP [27] has some limitations. It has low accuracy in predicting new drugs. Because the relationship prediction relies on the known information in the training set, which ignores the negative samples, the existing datasets are not accurate in the samples' categories classification. Training the classifier on this basis will lead to a deviation from the predicted results. In addition, according to the formulas of WNN, if a target has more drugs interacting with it, the prediction score of the target is higher, and this target is easier to be considered to have an interaction with new drugs. If there are fewer known interaction drugs at a certain target, the prediction score of this target will be lower. It will predict that there is no interaction relationship between this target and the new drug. At present, the cognition of drug-to-target interaction is not comprehensive, and a large number of interactions have not been approved. Therefore, only predicting the interaction of new drugs based on the known interaction will cause errors in the prediction results. For the sake of decreasing the prediction deviation caused by the existing dataset, the proposed method selected some drug-to-target pairs with possible interactions from unlabeled samples and revised the dataset to reduce the error. It improves the prediction performance of WNN-GIP.

## 3. Weighted Fusion of Multisource Information

**Figure 2:**
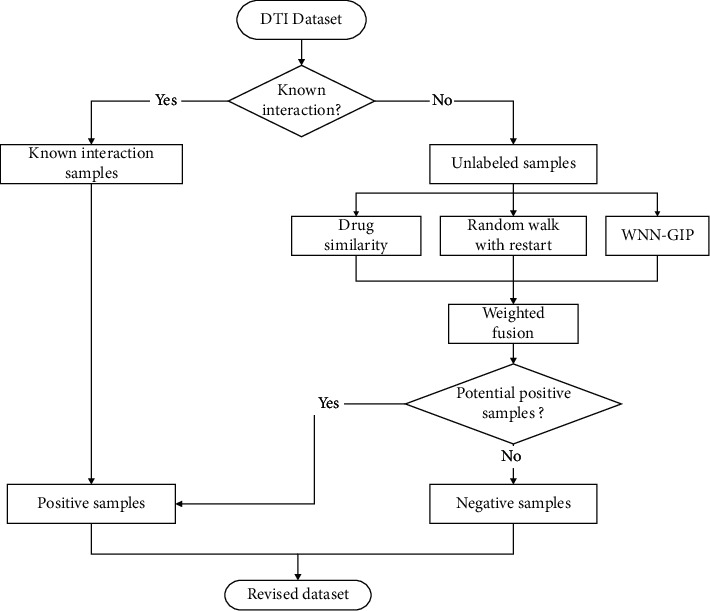
Flow chart of multisource information weighted fusion.

Prediction methods based on drug similarity, random walk with restart, and WNN-GIP have their own advantages. The methods based on drug similarity can make better use of the structural similarity between drugs to predict their interactions. Random walk with restart can integrate multiple networks, which makes full use of the correlation between nodes to predict. WNN-GIP can predict new drugs with low computational complexity. For the sake of combining the advantages of the abovementioned three methods, decreasing the computational complexity, and improving the prediction accuracy, a drug-to-target prediction method based on multisource information weighted fusion is proposed. The flow chart is shown in Figure 2.

**Figure 3:**
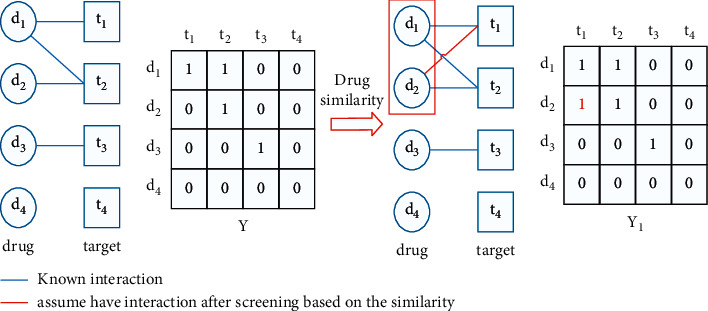
Screening based on drug similarity.

In this paper, based on the chemical structure information of the KEGG LIGAND database [37], the drug similarity is calculated according to the size of the common substructure [38]. It is known that drugs with high similarity may have identical targets [39]. The higher the similarity is, the higher the possibility of having the same target is. Therefore, according to the similarity matrix of drugs, some drugs with high similarity can be selected. For example, drug *d*_1_ has a high similarity with drug *d*_2_. It is known that drug *d*_1_ interacts with target *t*_1_, but the interaction between drug *d*_2_ and target *t*_1_ is unknown. Therefore, according to the abovementioned conditions, we can assume that drug *d*_2_ interacts with target *t*_1_, but it has not been proved yet. In this paper, drug-to-target pairs are screened by using the abovementioned three methods for the revision of the drug-to-target interaction matrix. The details are as follows:

According to the selected drugs with high similarity and the abovementioned hypothesis, some pairs with possible interactions are selected, and the existing interaction matrix is revised to get a new interaction matrix. Using revised datasets for prediction can reduce the false-negative error caused by treating unlabeled samples as negative samples. The process of interaction matrix revision based on drug similarity is shown in Figure 3.

In Figure 3, the circle represents the drug, the square represents the target, the line between the circle and the square represents the interaction between the drug and the target, Y is the original interaction matrix, and Y_1_ represents the modified interaction matrix after similarity screening.

The abovementioned selected pairs are added to the positive samples in the training set, and the transition matrix of the random walk is represented by *W*, which could be expressed as follows:

(5) $$\begin{array}{c} W=\left[\begin{array}{cc} W_{\text{TT}} & W_{\text{TD}}\\ W_{\text{DT}} & W_{\text{DD}} \end{array}\right], \end{array}$$

where elements denote the probability of transferring from network to network, *W*_TT_ denotes target to target, *W*_DD_ represents drug to drug, *W*_TD_ means target to drug, and *W*_DT_ is drug to target.

The random walk process in a heterogeneous network can be written as follows:

(6) $$\begin{array}{c} p_{t+1}=\left(1-c\right)W^{T}p_{t}+cp_{0}, \end{array}$$

where *p*_*t*_ denotes the probability after iterating *t* times. The parameter *c* means the restart probability. *p*_0_ is the starting probability vector and can be expressed as follows:

(7) $$\begin{array}{c} p_{0}=\left[\begin{array}{c} \left(1-\eta \right)u_{0}\\ \eta v_{0} \end{array}\right], \end{array}$$

where *u*_0_ and *v*_0_ denote the initial probabilities of the target and drug network, respectively. The parameter *η* ∈ (0,1) weights the importance of the two seed nodes. After some steps, *p*_*t*_ will converge to vector *p*_*∞*_, where $p_{\infty }=\left[\begin{array}{c} u_{\infty }\\ v_{\infty } \end{array}\right]$. When the Frobenius norm gap between *p*_*t*_ and *p*_*t*+1_ does not exceed 10^−10^, the iteration stops. The steady-state probability vector *p*_*∞*_ denotes the probability of interaction, so the revised matrix *Y*_2_ can be obtained.

In the training set, the abovementioned selected pairs are added to the positive samples, and *Y*_3_ is obtained by WNN-GIP. If the drug-to-target interaction matrix *Y*_1_, *Y*_2_, and *Y*_3_ obtained by the abovementioned three methods are averaged directly, the method with poor prediction effect will have a greater impact on the prediction results. Therefore, our method uses the weighted fusion method to obtain the final drug-target interaction matrix *Y*_final_, that is,

(8) $$\begin{array}{c} Y_{\text{final}}=\sum_{i=1}^{n}\alpha_{i}Y_{i}, \end{array}$$

where parameter *α*_*i*_ means the weight of *Y*_*i*_ obtained by each method and the value range from 0 to 1 and ∑_*i*=1_^*n*^*α*_*i*_=1. *Y*_*i*_ denotes the revised matrix, and *Y*_final_ denotes the drug-target interaction matrix after weighted fusion. In this paper, three revised matrices are obtained by screening with three methods, so *n* is equal to 3.

The weight of each method represents its contribution to the results, and the weight is determined by its prediction effect. The method with good effect contributes a lot to the result, and the corresponding weight is also large. The final matrix *Y*_final_ is intended for predicting interactions by the BLM. After the revised drug-to-target interaction matrix, the number of positive samples increases, which decreases the sparsity of data and, thereby, greatly improves the predictive ability.

## 4. Experiment

### 4.1. Datasets and Evaluation Metrcis

In this paper, we adopt datasets summarized in the literature [40]. The datasets contain four protein families of known drug-target datasets, which include enzymes (Es), ion channels (ICs), G-protein-coupled receptors (GPCRs) [41], and nuclear receptors (NRs). Each dataset contains three matrices: the drug similarity matrix, target protein similarity matrix, and drug-to-target interaction matrix. Matrix *Y*_*n*×*m*_ denotes the interaction, where *n* and *m* are the number of drugs and targets, respectively. If drug *d*_*i*_ interacts with target *t*_*j*_, then *y*(*i*, *j*)=1; otherwise, *y*(*i*, *j*)=0. The statistical information is shown in Table 1. It can be found that there are few known interactions in existing datasets.

The pairs with known interactions only account for a small fraction of the available data and most relationships are unknown, which leads to a small number of positive samples in the current datasets. The proportion of unlabeled samples is large, and the dataset is unbalanced. If only one evaluation index is used to evaluate our method, it is not comprehensive enough. Therefore, four basic indexes of accuracy, sensitivity, specificity, and precision are intended for assessing the model capability.

For better describing the superiority of the proposed method, the receiver operating characteristic curve (ROC) is also intended for assessing the capability of the DTI method. The ROC curve was drawn with true positive rate as the ordinate and false-positive rate as the abscissa. The closer the ROC curve gets to the top left corner, the higher the accuracy of the DTI prediction method. The ROC curve combines sensitivity and specificity with a graphic method, which can simply and intuitively analyze the accuracy of the experimental method. The values of AUC and AUPR are also given. AUC is the area under the ROC curve. AUC is greater than 0 and less than 1 [42]. The larger the AUC is, the higher the accuracy of the DTI prediction method is. AUPR is the area under the precision-recall (PR) curve. The value of AUPR is between 0 and 1. The higher the value of AUPR is, the higher the prediction accuracy is [43].

### 4.2. Experimental Results

To prove the validity of the dataset revised by multisource information fusion in the proposed method, we compared the proposed method with the BLM-NII method in accuracy, sensitivity, specificity, and precision. A 10-fold cross-validation is used in this paper. When calculating, all prediction results are sorted. The top 1% pairs are taken as positive samples. The accuracy, sensitivity, specificity, and precision of the prediction results can be acquired by comparing the prediction results with known datasets.  Table 2 shows the comparative results of the two methods. The red font in the table indicates the best experimental results.

From Table 2, we can know that the objective evaluation index of the proposed method is the highest among the four datasets. Compared with BLM-NII, the accuracy, sensitivity, and accuracy of the proposed method in the NR dataset are improved by 1.4%, 14.28%, and 6.67%, respectively. According to the consequence in Table 2, we can find that multisource information fusion can improve the performance of the BLM-NII model in all aspects. Especially, among the four datasets, the proposed method in the NR dataset has the largest improvement range, which shows that our method has excellent capability even in small sample datasets.

**Figure 4:**
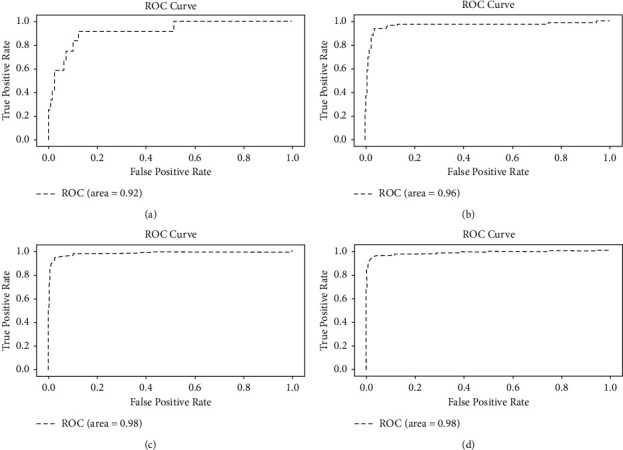
ROC of the proposed method in each dataset. (a) ROC in NR. (b) ROC in GPCR. (c) ROC in IC. (d) ROC in E.

To analyze the capability of our method more intuitively, Figure 4 shows the ROC of our method when tested on four datasets.  Figure 4(a) is the ROC obtained by the proposed method in the NR dataset. Because there are not many samples in the NR dataset, the curve is not very smooth and the area under the curve reaches 0.92.  Figure 4(b) is the ROC obtained in the GPCR dataset. Compared with the curve in the NR dataset, the capability of the proposed method in the GPCR dataset is superior with higher accuracy. According to Figures 4(c) and 4(d), the AUC in the IC dataset and E dataset both reach 0.98, which indicates our method performs superior in the dataset with more samples. More positive samples are helpful to promote the prediction capability.

To state the validity of the multisource information fusion method, we compare the proposed method to the prediction results when the drug-target dataset is revised by a single method. These methods are as follows: (1) SIM: selected pairs based on drug similarity; (2) RS: selected pairs based on the random walk with a restart; and (3) WS: selected pairs based on WNN-GIP. The objective evaluation indicators adopted in this paper are AUC and AUPR. The experimental results are demonstrated in Table 3. The red font part represents the best experimental result among several methods, and the blue font part represents the suboptimal experimental result. Tables 3–8 are represented in the same way.

In Table 3, SIM, RS, and WS, respectively, represent the experimental results when the initial interaction matrix is revised only by the drug similarity, random walk with restart, and WNN-GIP. Observing Table 3, it can be found that the weighted fusion method of multisource information proposed in this paper has obtained the highest AUC and AUPR in four datasets. Therefore, the fusion of multisource information can combine the advantages of each method, and the fusion method can effectively improve the accuracy of prediction. The experimental results show that the weighted fusion method of multisource information has superior capability to the single screening method when revising the dataset.

To state the validity of the weighted fusion method, the fusion method is replaced by the average fusion method (AVE) and the voting fusion method (VOTE). The results are displayed in Table 4.

AVE stands for the experimental results obtained by averaging DTI matrices *Y*_1_, *Y*_2_, and *Y*_3_ by the abovementioned three methods as the final revision matrix. VOTE represents the experimental result when the abovementioned three matrices are processed by the majority voting method. From Table 4, it can be found that AUC and AUPR obtained by the weighted fusion method are both the highest. Also, either the average or the voting method will greatly reduce the prediction accuracy. The weighted fusion method can assign different weights depending on the results of the three methods to achieve superior fusion effects.

To certify the availability of our method, we compared the proposed method with several state-of-the-art methods, whichare as follows: (1) NetLapRLS [44]: a DTI prediction method based on semisupervised learning; (2) BLM-NII [33]: a DTI prediction method based on BLM improved by the neighbor interaction profile inferring; (3) WNN-GIP [27]: a DTI prediction method based on GIP improved by a weighted nearest neighbor; (4) ALADIN [34]: a DTI prediction method based on advanced local drug-to-target interaction prediction technique; and (5) MOLIER [35]: a DTI prediction method based on a modified linear regression model.

Tables 5–8 are the results of DTI prediction in NR, IC, GPCR, and E datasets by the abovementioned methods and our method, respectively.

In Table 5, the AUC and AUPR of our method are the highest in the NR dataset, which indicates that our method has superior prediction ability even in datasets with few samples. Compared with ALADIN and NetLapRLS, the prediction effect of our method is obviously improved, which shows that it can reduce the influence of sample numbers.

In Table 6, the AUC and AUPR of our method are also the highest in the IC dataset, which shows that our method has achieved high prediction accuracy. The proportion of known interactions in this dataset is the highest among the four datasets, which is the key for the proposed method to achieve excellent performance. More known interactions can help predict relationships in the network.

Table 7 shows that the proposed method has top values in AUC and the suboptimal AUPR in the GPCR dataset. This is mainly because the average number of interactions in the E and IC datasets is larger than that in the GPCR dataset. That is to say, in the training phase, the proportion of positive samples in the E and IC datasets is much higher than that in the GPCR dataset. This is beneficial to get a classifier model with superior prediction capability. Therefore, AUPR can obtain higher values in the E and IC datasets. The ratio of positive samples in the GPCR dataset is small, so the display of the proposed method in the GPCR dataset is slightly poor.

Table 8 shows that our method obtains the top values in AUC and AUPR in the E dataset. The performance of ALADIN, MOLIER, and the proposed method are all improved on the basis of BLM-NII. The results show that the AUC and AUPR of the proposed method are better than those of the first two methods in most cases, especially in the small sample of the NR dataset. In general, the capability of the proposed method is good, but there is still room for improvement, and AUPR needs to be further improved.

## 5. Conclusions

In this paper, a DTI prediction method based on the weighted fusion of multisource information is proposed. In this method, the samples with unknown interaction relationships are regarded as unlabeled samples. The samples which may have interaction but have not been verified by experiments are screened out, and the original dataset is revised according to the screening results. According to the experimental results, we can find that the proposed weighted fusion method is more reasonable than the averaging and voting methods. The weighted fusion method increases the effectiveness and reliability of the screening results. Both the AUC and AUPR of the proposed method have achieved better results. However, the proposed method also has some limitations. It performs better in datasets with more samples, while the generalization ability will become worse in datasets with fewer samples. Especially for datasets with fewer positive samples, the prediction accuracy needs to be improved. It may be that the fusion model has brought some restrictions, and AUPR should be further improved. In the future, we can combine more biological information in prediction so that more drug-target pairs with known interactions can be introduced. Because more known relationships can reduce isolated nodes in the network, it is more helpful to predict edge relationships in the network. Meanwhile, we can further explore the fusion method. The goal is to find a fusion model that can be flexibly change to achieve a better fusion effect. Next, we can reduce the constraints brought by fusion to optimize the model.

## Figures and Tables

**Table 1:** Summary of the datasets.

Dataset	Drugs	Targets	Drug-target interactions	Unknown interactions
NR	54	26	90	1314
GPCR	223	95	635	20550
IC	210	204	1476	41364
E	445	664	2926	292554

**Table 2:** Comparison of accuracy, sensitivity, specificity, and precision between our method and BLM-NII.

Dataset	Method	Accuracy	Sensitivity	Specificity	Precision
NR	BLM-NII	91.66	71.43	92.70	33.33
	Ours	93.06	85.71	93.43	40.0
GPCR	BLM-NII	92.28	88.89	92.38	26.29
	Ours	92.75	96.83	92.62	28.64
IC	BLM-NII	93.28	92.22	93.32	35.90
	Ours	93.56	95.81	93.47	37.30
E	BLM-NII	90.81	92.83	90.79	8.76
	Ours	90.86	95.70	90.82	9.04

**Table 3:** Comparison of AUC and AUPR values between the proposed method and other single screening methods.

AUC/AUPR	NR	GPCR	IC	E
SIM	0.922/0.586	0.960/0.547	0.978/0.777	0.982/0.686
RS	0.909/0.567	0.936/0.483	0.976/0.830	0.972/0.687
WS	0.908/0.582	0.943/0.518	0.984/0.718	0.971/0.569
Ours	0.925/0.717	0.963/0.707	0.986/0.914	0.985/0.898

**Table 4:** Influence of different fusion methods on the prediction of drug-target interactions.

AUC/AUPR	NR	GPCR	IC	E
AVE	0.903/0.655	0.883/0.351	0.964/0.718	0.966/0.436
VOTE	0.897/0.616	0.894/0.400	0.970/0.759	0.964/0.437
Ours	0.925/0.717	0.963/0.707	0.986/0.914	0.985/0.898

**Table 5:** AUC and AUPR values of our method and several state-of-the-art methods in the NR dataset.

NR	AUC	AUPR
NetLapRLS	0.808	0.457
BLM-NII	0.903	0.655
WNN-GIP	0.871	0.584
ALADIN	0.664	0.310
MOLIER	0.911	0.683
Ours	0.925	0.717

**Table 6:** AUC and AUPR values of our method and several state-of-the-art methods in the IC dataset.

IC	AUC	AUPR
NetLapRLS	0.967	0.827
BLM-NII	0.964	0.718
WNN-GIP	0.953	0.653
ALADIN	0.980	0.875
MOLIER	0.983	0.912
Ours	0.987	0.914

**Table 7:** AUC and AUPR values of our method and several state-of-the-art methods in the GPCR dataset.

GPCR	AUC	AUPR
NetLapRLS	0.913	0.590
BLM-NII	0.882	0.350
WNN-GIP	0.930	0.498
ALADIN	0.946	0.680
MOLIER	0.952	0.753
Ours	0.963	0.707

**Table 8:** AUC and AUPR values of our method and several state-of-the-art methods in the E dataset.

E	AUC	AUPR
NetLapRLS	0.964	0.784
BLM-NII	0.966	0.436
WNN-GIP	0.957	0.748
ALADIN	0.966	0.822
MOLIER	0.985	0.897
Ours	0.986	0.898

## Data Availability

The DTI prediction test data used to support the findings of this study were supplied by http://web.kuicr.kyoto-u.ac.jp/supp/yoshi/drugtarget/ under license and so cannot be made freely available.
